# Hypovitaminosis D and morbidity in critical illness: is there proof beyond reasonable doubt?

**DOI:** 10.1186/cc13863

**Published:** 2014-05-08

**Authors:** Bala Venkatesh, Priya Nair

**Affiliations:** 1 Princess Alexandra Hospital, 199 Ipswich Road, Wooloongabba QLD 4102, Australia; 2 Intensive Care, The Wesley Hospital, 451 Coronation Drive, Auchenflower QLD 4066, Australia; 3 University of Queensland, Brisbane, St Lucia QLD 4072, Australia; 4 Intensive Care Unit, St Vincent’s Hospital, 41 Victoria Parade, Fitzroy VIC 3065, Australia; 5 University of New South Wales, High Street, Kensington NSW 2052, Australia; 6 Garvan Institute of Medical Research & George Institute for Global Health, 384 Victoria Street, Darlinghurst Sydney NSW 2010, Australia

## Abstract

Vitamin D is recognized to have important actions outside its well-recognized role in musculoskeletal health. These include antimicrobial action, anti-inflammatory, and cardio-protective properties. A high prevalence of vitamin D deficiency and its association with adverse clinical outcomes have now been widely documented in observational studies in the critically ill. These studies of association, however, do not necessarily imply causation, as vitamin D deficiency may be merely a marker of higher illness severity and consequently poorer outcomes. This issue can be clarified only by undertaking high-quality randomized controlled trials of vitamin D supplementation in this vulnerable population.

## Introduction

In the previous issue of *Critical Care*, Amrein and colleagues [1] report a single-center observational study of 655 surgical and non-surgical critically ill patients over a 2-year period and describe the association between vitamin D status and seasonality and mortality in critical illness. Vitamin D, traditionally considered important for the maintenance of calcium homeostasis and musculoskeletal health, is now widely recognized for its extra-skeletal ‘pleiotropic effects’. Over the past decade, data from biochemical and molecular genetic studies indicate that vitamin D - particularly its active form, 1,25-dihydroxy cholecalciferol (1,25-dihydroxy-D3) - has a much wider role. These pleiotropic effects include potentiation of antimicrobial action and cardio-protective and immunomodulatory effects. The discoveries that most tissues and cells in the body have vitamin D receptors and that several possess the 1-α hydroxylase enzyme have provided novel insights into the pleiotropic actions of this vitamin [2].

## Hypovitaminosis D: Prevalence and associations with clinical outcome

Vitamin D is synthesized in the skin and then undergoes 25-hydroxylation in the liver to 25-hydroxy-D3 followed by 1α-hydroxylation, especially but not exclusively in the kidneys, to 1,25-dihydroxy-D3. Based on optimal bone health, a widely used definition considers a serum 25-hydroxy-D3 of less than 20 ng/mL (50 nmol/L) to be a vitamin D deficiency, 20 to 30 ng/mL (50 to 75 nmol/L) to be insufficient, and more than 30 ng/mL (75 nmol/L) to be normal. Although renal 1α-hydroxylase activation may be important for circulating 1,25-dihydroxy-D3 levels, locally 1,25-dihydroxy-D3 formed by tissue 1α-hydroxylase is critical in mediating the pleiotropic actions of vitamin D. Vitamin D sufficiency is required for both the endocrine and paracrine arms of the axis to function effectively.

There is increasing evidence that vitamin D deficiency is common in hospitalized patients and particularly in the critically ill. Hypovitaminosis D has been associated with increased incidence of a number of autoimmune conditions and adverse outcomes in cardiac transplant recipients and the general population [3]. Although vitamin D insufficiency does not pose immediate health hazards in otherwise healthy individuals, it may be detrimental during critical illness, as the circulating vitamin D pool functions as an important reservoir for local conversion to the active metabolites at tissue level during stress [4].

Global interest in the significance of vitamin D deficiency in critical illness was triggered in 2009 following a report by Lee and colleagues [5], who noted a previously under-recognized high prevalence of hypovitaminosis D. Only 7 % of patients were vitamin D-sufficient, and a predicted mortality was three times higher in deficient patients. Subsequently, a number of studies in critically ill patients have reported a prevalence ranging from 38 % to 100 %, which is about 50 % higher than that for patients in general medical wards.

Several observational studies in critically ill patients have also demonstrated an association between vitamin D deficiency/insufficiency and poor outcomes. These include increases in illness severity and risk of death, ICU length of stay [6-8], ventilator requirement [9], rates of infection (including ventilator-associated pneumonia) [10], blood culture positivity [11], organ dysfunction, and short-term and long-term hospital mortality [5,9,11]. Furthermore, ICU and hospital costs were found to be higher for vitamin D-deficient patients.

In this context, the study findings of Amrein and colleagues are in accord with previously published data. Vitamin D levels were categorized by month-specific tertiles (high, intermediate, and low) to reflect seasonal variation of serum 25-hydroxy-D3 levels. There was a high prevalence of vitamin D deficiency and insufficiency. Adjusted hospital mortality was significantly higher in patients in the low and intermediate compared with those in the high tertiles. No sepsis-related fatality was observed in the highest month-specific tertile or in vitamin D sufficiency. The authors concluded that low 25-hydroxy-D3 status is significantly associated with all-cause and sepsis mortality in the critically ill and suggested that interventional studies are warranted to study the effect of vitamin D supplementation on outcome in this population. However, the indications for vitamin D assays in these patients were unclear; it was a convenience sample resulting in a selection bias as only patients who had vitamin D assays performed were chosen. There were no differences in bacteremia between survivors and non-survivors. Data on inflammatory markers were not reported. Moreover, vitamin D levels are influenced by fluid shifts and diurnal variability, throwing into doubt the significance of a single measurement [12,13].

Despite these limitations, the study adds to the burgeoning volume of literature on the prevalence of hypovitaminosis D in critically ill patients and the resulting potential for adverse outcomes. Where do we go from here? There have been calls for universal vitamin D supplementation in critically ill patients. Given the present evidence, this would be premature as a number of caveats still remain.

The criteria for definitions of vitamin D sufficiency are based on bone health; the optimal level for pleiotropy remains unclear. Moreover, little data exist on the bio-active form, 1,25-dihydroxy-D3, in critical illness and association with outcomes. Reports of supra-physiological levels of 1,25-dihydroxy-D3 following inflammatory response in critically ill patients [12] and, more recently, evidence of extra-renal production of 1,25-dihydroxy-D3 [14] have been published. Furthermore, the high degree of protein binding of 25-hydroxy-D3 and consequently the relevance of free levels, particularly in the context of altered pharmacokinetics associated with critical illness, remain to be elucidated.

## Conclusions

The observational studies available do not suggest causation, and hypovitaminosis D may be simply a marker of poor health, which predicts patients who would have poorer outcomes. Reports that supra-physiological levels of 25-hydroxy-D3 may be associated with adverse outcomes are also emerging [15]. The only means to clarify the role for supplementation is to conduct appropriate dose-response studies followed by a high-quality placebo-controlled randomized trial of vitamin D supplementation in this vulnerable population. Vitamin D is inexpensive and, if it is shown to be beneficial, there is no barrier to rapid implementation into routine clinical practice worldwide.

## Competing interests

The authors declare that they have no competing interests.
